# Anti-Hypertensive Effects of Acacia Polyphenol in Spontaneously Hypertensive Rats

**DOI:** 10.3390/ijms19030700

**Published:** 2018-03-01

**Authors:** Nobutomo Ikarashi, Takahiro Toda, Yusuke Hatakeyama, Yoshiki Kusunoki, Risako Kon, Nanaho Mizukami, Miho Kaneko, Sosuke Ogawa, Kiyoshi Sugiyama

**Affiliations:** Department of Clinical Pharmacokinetics, Hoshi University, 2-4-41 Ebara, Shinagawa-ku, Tokyo 142-8501, Japan; t.toda@thu.ac.jp (T.T.); xdxexexnxfxoxvxwxaxnxdxsx@gmail.com (Y.H.); p16kusunoki@hoshi.ac.jp (Y.K.); r-kon@hoshi.ac.jp (R.K.); m1616@hoshi.ac.jp (N.M.); m1703@hoshi.ac.jp (M.K.); ogawa@mimozax.co.jp (S.O.); sugiyama@hoshi.ac.jp (K.S.)

**Keywords:** acacia polyphenol, hypertension, superoxide dismutase, nicotinamide adenine dinucleotide phosphate oxidase

## Abstract

We have previously demonstrated that acacia polyphenol (AP) exerts strong anti-obesity, anti-diabetic, and anti-atopic dermatitis effects. In the present study, we investigated the anti-hypertensive effects of AP. Spontaneously hypertensive rats (SHR) with hypertension and control Wistar Kyoto rats (WKY) were used. WKY and SHR were fed AP-containing food or AP-free food (control group) ad libitum for 4 weeks, and their blood pressures were measured. After AP administration, both systolic and diastolic blood pressures were significantly lower in the SHR group than in the control group. There were no differences in the systolic or diastolic blood pressure of WKY between the AP group and the control group. Angiotensin-converting enzyme (ACE) activity, *nicotinamide adenine dinucleotide phosphate* (*NADPH*) *oxidase* expression, and superoxide dismutase (SOD) activity in SHR kidneys were not altered by AP administration. Blood SOD activity in SHR was significantly higher in the AP group than in the control group. AP exerts anti-hypertensive effects on hypertension but has almost no effect on normal blood pressure. The anti-hypertensive effects of AP may be related to the anti-oxidative effects of increased blood SOD activity.

## 1. Introduction

Acacia is an evergreen tree belonging to the genus *Acacia* in the legume family, and it is widely distributed throughout the Australian and African continents. *Acacia catechu* duramen (heartwood) extract is called gambir. In Japan and China, gambir has long been used as an astringent and antibacterial agent. Polyphenol-rich aqueous extract from the bark of *Acacia mearnsii* De Wild. (Acacia polyphenol; AP), which is widely distributed throughout South Africa, has been used to eliminate wine sediment in Europe. In addition, Australian Aborigines eat the young leaves and beans of *Acacia mearnsii* De Wild.

We have previously demonstrated that AP exerts anti-obesity and anti-diabetic actions by altering the expression of genes associated with the inhibition of obesity and diabetes in skeletal muscle, liver, and white adipose tissues [1]. In addition, we have shown that AP inhibits lipase and glucosidase activities resulting in the inhibition of increased plasma triglyceride and glucose levels [2]. We have also found that AP exerts anti-atopic dermatitis actions [3]. Thus, AP possesses various pharmacological effects.

In recent years, polyphenols have been reported to have anti-hypertension effects [4,5]. AP contains significant amounts of polyphenols, including monometric flavan-3-ols, such as catechin and procyanidin B-2, and proanthocyanidin oligomers. The acacia proanthocyanidin oligomers are mainly composed of 5-deoxyflavan-3-ol units, such robinetinidol and fisetinidol and their chemical properties are different from those of many other proanthocyanidins [6]. Accordingly, similarly to these polyphenols, AP may exert anti-hypertensive effects, as well as anti-obesity, anti-diabetic, and anti-atopic dermatitis actions. Therefore, in this study, the effects of AP on hypertension were investigated by using spontaneously hypertensive rats (SHR) and control Wistar Kyoto rats (WKY). The mechanism of the anti-hypertensive effects of AP was also examined.

## 2. Results

### 2.1. Food Intake and Body Weight

**Figure 1:**
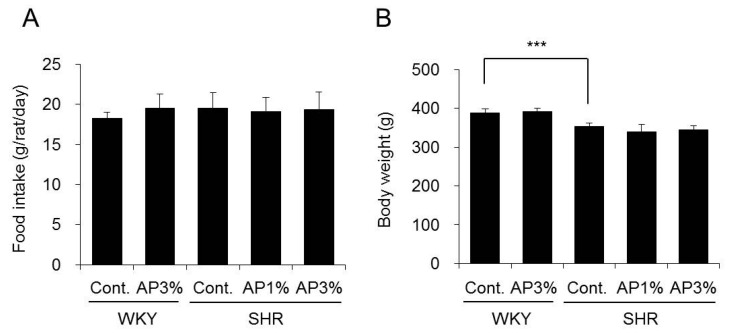
Food intake and body weight. A powdered diet containing AP or a diet without AP (control group) was fed to WKY and SHR. After 4 weeks, food intake (**A**) and body weight (**B**) were determined. Body weight is presented as the mean ± standard deviation (S.D.) (*n* = 5). Food intake for each cage (5 rats/cage) in each group was determined, and the mean food intake by day was calculated. Tukey’s test; *** *p* < 0.001.

AP was administered to the WKY and SHR groups for 4 weeks, and food intake and body weight were measured. Treatment with AP did not change the food intake or body weight of WKY. There were also no changes in the food intake or body weight after treatment with AP in SHR (Figure 1).

### 2.2. Heart Weight and Heart Rate

**Figure 2:**
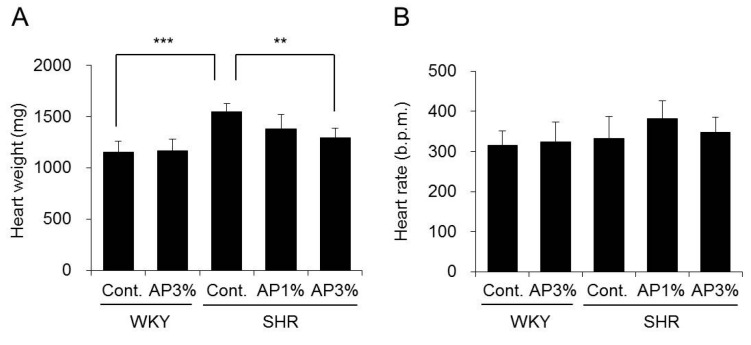
Heart weight and heart rate. A powdered diet containing AP or a diet without AP (control group) was fed to WKY and SHR. After 4 weeks, heart weight (**A**) and heart rate (**B**) were measured. The data are presented as the means ± S.D. and were obtained from 5 rats per group. Tukey’s test; ** *p* < 0.01, *** *p* < 0.001.

AP was administered to WKY and SHR for 4 weeks, and their heart weight and heart rate were measured. Administration of AP did not change the heart weight of WKY, but the heart weight of SHR decreased in a dose-dependent manner. There was a significant difference between SHR fed food with 3% AP and SHR fed normal food (Figure 2A). There were no changes in heart rate after treatment with AP in WKY and SHR (Figure 2B).

### 2.3. Systolic Blood Pressure (SBP) and Diastolic Blood Pressure (DBP)

**Figure 3:**
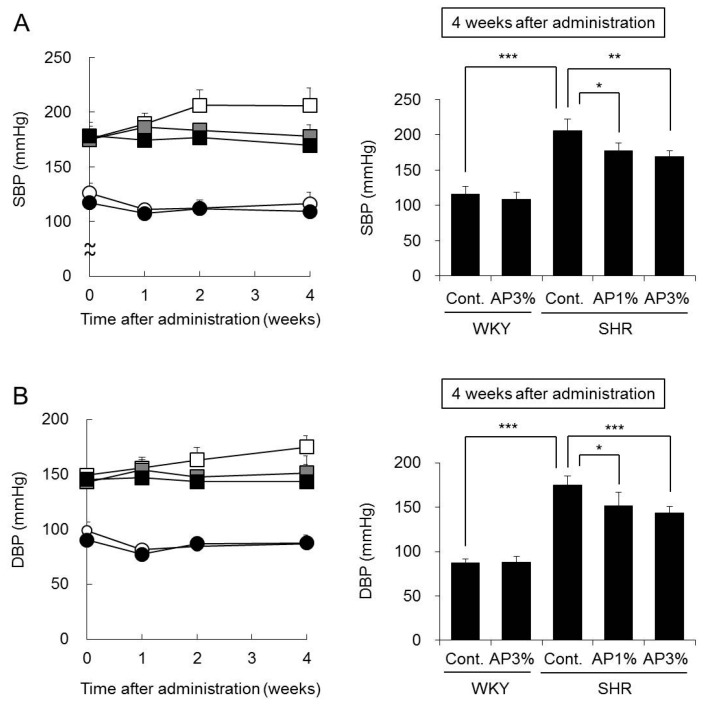
Systolic blood pressure (**A**) and diastolic blood pressure (**B**). A powdered diet containing AP or a diet without AP (control group) was fed to WKY and SHR. SBP (**A**) and DBP (**B**) were measured every week. The data are presented as the means ± S.D. and were obtained from 5 rats per group. White circle: WKY-control group; black circle: WKY-AP 3%-treated group; white square: SHR-control group; gray square: SHR-AP 1%-treated group; and black square: SHR-AP 3%-treated group. Tukey’s test; * *p* < 0.05, ** *p* < 0.01, *** *p* < 0.001.

AP was administered to WKY and SHR for 4 weeks, and their SBP and DBP were measured. The SBP and DBP of WKY fed food with 3% AP were almost the same as those in WKY fed normal food (Figure 3). The SBP and DBP of SHR were higher than those of WKY throughout the experimental period. Compared with the SBP of the controls, the SBP of SHR treated with AP decreased from the first week of treatment. The SBP of SHR treated with AP for 4 weeks decreased significantly depending on the dose of AP. DBP and SBP showed similar results (Figure 3).

### 2.4. Angiotensin-Converting Enzyme (ACE) Activity in the Kidney

ACE is crucial in the regulation of the renin-angiotensin system; it cleaves angiotensin-I, thus yielding angiotensin-II, and hydrolyzes and inactivates the vasodilator peptide bradykinin [7]. ACE inhibition is an important therapeutic approach in the treatment of high blood pressure. Therefore, the influence of AP on ACE activity in the cortex and medulla of the kidney, which contain high amounts of ACE, was examined.

**Figure 4:**
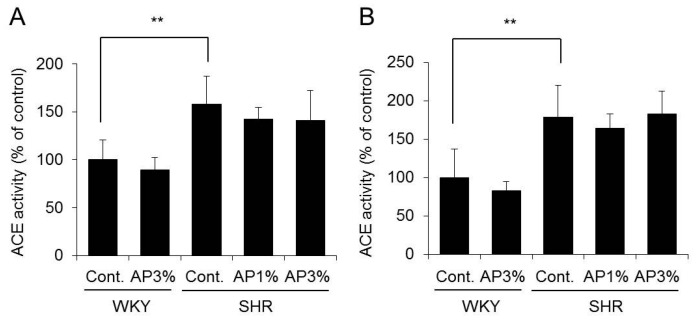
ACE activity in the kidney. A powdered diet containing AP or a diet without AP (control group) was fed to WKY and SHR. ACE activity in the cortex (**A**) and medulla (**B**) in the kidney was measured. The data are presented as the means ± S.D. and were obtained from 5 rats per group. Tukey’s test; ** *p* < 0.01.

Administration of AP to WKY did not change ACE activity in the kidney. In addition, ACE activity in the cortex and medulla of the kidney did not differ between SHR receiving AP and the controls (Figure 4).

On the basis of these results, AP did not affect ACE activity in the kidney.

### 2.5. Expression of Nicotinamide Adenine Dinucleotide Phosphate (NADPH) Oxidase in the Kidney

Superoxide anion is a reactive oxygen species (ROS) generated by NADPH oxidase [8]. ROS is associated with the development of hypertension [9], and inhibition of the expression and activity of NADPH oxidase in the kidney inhibits the development of hypertension [10]. Therefore, the influence of AP on the expression of *NADPH oxidase* in the kidney was examined.

**Figure 5:**
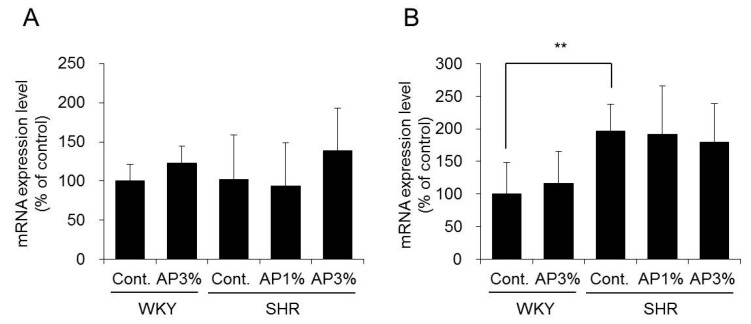
The mRNA expression levels of *NADPH oxidase* in the kidney. A powdered diet containing AP or a diet without AP (control group) was fed to WKY and SHR. The mRNA expression levels of *p22* (**A**) and *p47* (**B**) in the kidney were measured with real-time RT-PCR and normalized to *18S rRNA*. The results were obtained by setting the means of the control group to 100%. The data are presented as the means ± S.D. and were obtained from 5 rats per group. Tukey’s test; ** *p* < 0.01.

The mRNA expression levels of the *p22* and *p47* subunits of *NADPH oxidase* have been analyzed [11,12]. Treatment with AP did not change the mRNA expression levels of *p22* and *p47* in the kidney of SHR (Figure 5).

On the basis of these results, AP did not affect the expression of *NADPH oxidase* in the kidney.

### 2.6. Superoxide Dismutase (SOD) Expression and Activity in the Kidney

SOD is a well-known anti-oxidant enzyme that converts superoxide anion to hydrogen peroxide [13,14]. As previously mentioned, ROS are related to the development of hypertension. Therefore, the influence of AP on the expression and activity of SOD in the kidney was examined.

**Figure 6:**
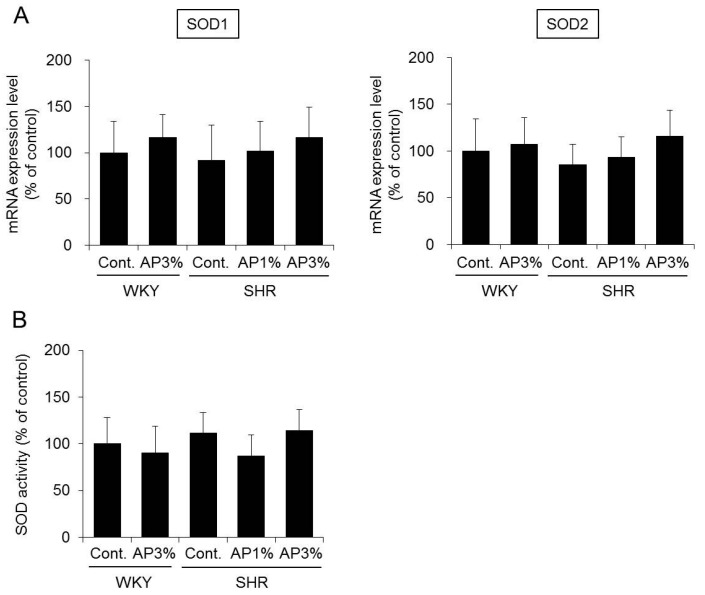
SOD expression (**A**) and activity (**B**) in the kidney. A powdered diet containing AP or a diet without AP (control group) was fed to WKY and SHR. (**A)** The mRNA expression levels of *SOD1* and *SOD2* in the kidney were measured with real-time RT-PCR and normalized to *18S rRNA*. (**B**): SOD activity in the kidney was measured. The results were obtained by setting the means of the control group to 100%. The data are presented as the means ± S.D. and were obtained from 5 rats per group.

SOD has three known isoforms [13,14]. Among these isoforms, the mRNA expression levels of *SOD1* and *SOD2*, which are important in SOD activity [13], were analyzed herein. Treatment with AP did not change the mRNA expression of *SOD1* in the kidney of SHR. There was also no difference in the expression of *SOD2* between the control group and the AP treatment group (Figure 6A). Measurement of the SOD activity in the kidney also did not differ between the control group and the AP treatment group (Figure 6B).

On the basis of these results, AP did not affect the expression and activity of SOD in the kidney.

### 2.7. SOD Activity in the Blood

Increased SOD activity in the blood reduces ROS in the blood and inhibits hypertension [15]. Therefore, the mechanism of the anti-hypertensive effects of AP was analyzed by measuring SOD activity in the blood.

**Figure 7:**
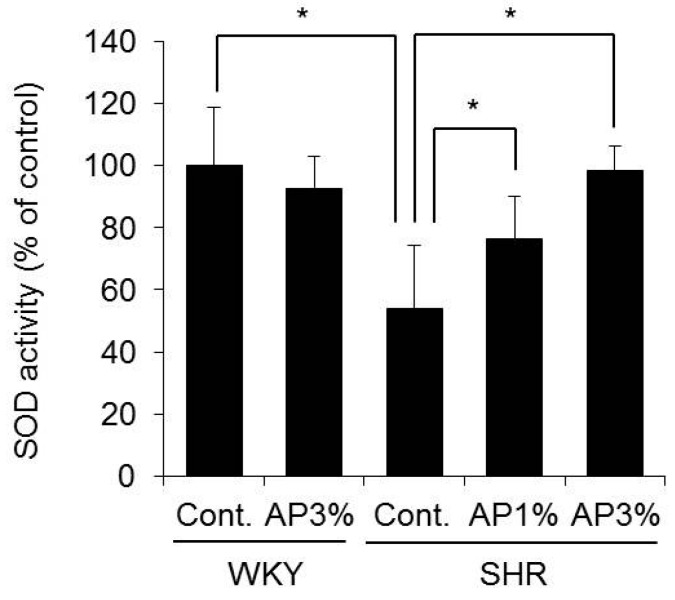
SOD activity in the blood. A powdered diet containing AP or a diet without AP (control group) was fed to WKY and SHR. Plasma SOD activity was measured. The results were obtained by setting the means of the control group to 100%. The data are presented as the means ± S.D. and were obtained from 5 rats per group. Tukey’s test; * *p* < 0.05.

SOD activity in the blood significantly decreased in SHR compared with that in WKY. Compared with controls, SHR showed increased SOD activity in the blood after treatment with AP in a dose-dependent manner (Figure 7).

Therefore, AP may inhibit increases in blood pressure by increasing SOD activity in the blood.

## 3. Discussion

According to our previous study, AP exerts anti-obesity, anti-diabetic, and anti-atopic dermatitis actions when it is administered to mice in a diet containing 1.5–5% AP [1,3]. In this study, rats received a diet containing 1% or 3% AP for 4 weeks, and we examined the effect of AP on hypertension. The body weight and food intake in AP-treated rats were similar to those in control-treated rats (Figure 1), and no toxicity was observed in rats treated with AP.

The SHR strain is derived from the WKY strain, and these animals are widely used as a model for hypertension in humans [16,17,18]. The blood pressure of SHR increased with age, and treatment with AP significantly inhibited this increase in blood pressure (Figure 3). Continued hypertension generally burdens the heart and causes myocardial hypertrophy. The heart weight in SHR is greater than that in WKY [10]. This finding was also observed in the present study, but AP significantly inhibited cardiac hypertrophy in SHR (Figure 2A), possibly because treatment with AP inhibited an increase in blood pressure and alleviated the burden on the heart. Myocardial fibrosis and cardiomyocyte size were not histologically evaluated in the current study. To date, it is known that there is a correlation between increase in heart weight and onset of myocardial fibrosis or cardiomyocyte [19,20]. Therefore, it is possible that AP prevents myocardial fibrosis or cardiomyocyte, and this point it to be studied in the future.

Next, we asked why AP inhibits increased blood pressure in SHR. Angiotensin-II plays an important role in increased blood pressure in SHR [9]. Angiotensin-II is produced by ACE in the kidney, and ACE activity inhibitors occupy an important position among anti-hypertensive treatments. Various natural substances containing polyphenol have been reported to inhibit ACE activity in the kidney [21]. Therefore, the influence of AP on ACE activity in the kidney was examined. AP caused almost no change in ACE activity SHR kidneys (Figure 4). ACE activity inhibitors decreased normal blood pressure not only in SHR but also in WKY [22,23]. However, AP did not decrease normal blood pressure in WKY (Figure 2). Our results suggest that AP does not possess ACE inhibitory activity. Angiotensin receptor inhibitors and calcium antagonists used in clinical settings also decrease the normal blood pressure of WKY [24,25]. Accordingly, compared with angiotensin receptor inhibitors and calcium antagonists, AP may also decrease the blood pressure of SHR through a different mechanism.

ROS are substances in the body that play an important role in the regulation of blood pressure. ROS are also involved in the development of hypertension in SHR [26]. Accordingly, substances that decrease the production and activity of ROS inhibit increased blood pressure. In a recent report, the anti-oxidative effects of polyphenols have been found to inhibit hypertension. For example, green coffee bean extract has been found to inhibit hypertension in SHR, an effect attributed to scavenging by ROS [27]. Azuki bean polyphenol and green coffee bean polyphenol do not decrease the blood pressure of healthy animals but do decrease the blood pressure of SHR [10,27]. AP possesses strong anti-oxidant activity. In addition, like other polyphenols, AP does not decrease the blood pressure of normal animals (Figure 2). Accordingly, the mechanism of the anti-hypertensive effect of AP is considered to be due to its anti-oxidative effects. Therefore, we examined whether the anti-hypertensive effects of AP might be attributable to the anti-oxidative effects of AP, especially effects on NADPH oxidase and SOD.

NADPH oxidase is involved in the generation of ROS. The p22 and p47 subunits of NADPH oxidase in the kidney are involved in an increase in blood pressure [28]. Treatment with AP did not change the expression of *NADPH oxidase* in SHR (Figure 5). Therefore, the anti-hypertensive effect of AP did not inhibit the expression/activity of NADPH oxidase; this result was different from those reported for Azuki bean polyphenol and wine polyphenol [10,29].

SOD has an oxidative effect, which converts ROS into hydrogen peroxide and oxygen. Mammals have three different SOD isoforms [13,14]. Copper/zinc SOD (Cu/ZnSOD or SOD1) is a cytosolic enzyme encoded by the *SOD1* gene and is the predominant SOD in most cells and tissues, accounting for 70–80% of total cellular SOD activity [13]. Manganese superoxide dismutase (MnSOD or SOD2) is a mitochondrial anti-oxidant enzyme encoded by the *SOD2* gene [13]. Extracellular copper/zinc SOD (SOD3), which is encoded by the *SOD3* gene, is expressed in only a limited number of tissues [14]. The expression and activity of SOD in the kidney are important in the development of hypertension. No changes occurred due to treatment with AP in a study of the expression levels of *SOD1* and *SOD2* and SOD activity in the kidney (Figure 6). In contrast, SOD activity in the blood in SHR treated with AP was significantly higher than that in rats treated with control (Figure 7). Pu-erh tea extract increases SOD activity in the blood [30]. Red raspberry extracts exert anti-hypertensive effects by increasing SOD activity in the blood [15]. On the basis of the present study results, similarly to the aforementioned polyphenol-containing extracts, AP exerts anti-oxidative effects by increasing SOD activity in the blood and inhibits increased blood pressure.

As described above, the anti-hypertensive effects of AP are related to the anti-oxidative effects of increased blood SOD activity. In addition to SOD activity, the expression levels and activity of endothelial nitric oxide synthase (eNOS) and inducible nitric oxide synthase (iNOS) in the kidney are involved in the onset of hypertension [26]. Azuki bean polyphenol inhibits hypertension via modulation of eNOS and iNOS expression levels in the kidney [31]. Although the expression levels of eNOS and iNOS were not analyzed in this study, the novel mechanisms of the anti-hypertensive effects of AP may potentially be elucidated by focusing on eNOS and iNOS in future studies.

In the present study, AP exhibited anti-hypertensive effects in cases of hypertension but exerted almost no effect in cases of normal blood pressure. The anti-hypertensive effects of AP might be attributed to an increase in SOD activity in the blood. Some components of AP (orally administered) are absorbed by the gastrointestinal tract and exert an anti-oxidative effect in the blood. AP may have clinical applications as a functional food if the details of the mechanism of the anti-hypertensive effect of AP and its active ingredient are clarified.

## 4. Materials and Methods

### 4.1. Materials

An RNeasy Mini Kit was purchased from Qiagen Inc. (Valencia, CA, USA). A High Capacity cDNA Synthesis Kit was purchased from Applied Biosystems (Foster City, CA, USA). iQ SYBR Green Supermix was purchased from Bio-Rad Laboratories (Hercules, CA, USA). Primers were purchased from Invitrogen Corp. (Tokyo, Japan). A SOD Assay Kit-WST was purchased from Dojindo (Kumamoto, Japan). All other reagents were of the highest commercially available grade.

### 4.2. Hot Water Extraction from Acacia Bark

AP (lot; D1) was donated by Mimozax Co., Ltd. (Hiroshima, Japan) and prepared according to Cutting’s methods [32]. Briefly, the powdered bark of South African *Acacia mearnsii* De Wild. was pulverized and extracted for 30 min in a 10-fold volume of hot water (100 °C) and then dried using a spray drier. The extract yield was approximately 35% (*w*/*w*). The polyphenol content of the present product was 79.0%, as measured by the Stiasny reaction. AP is a complex mixture of proanthocyanidins, including unique flavan-3-ol oligomers and polymers comprising 4 or 5 monomeric units, such as robinetinidol, fisetinidol, catechin, and gallocatechin. The average molecular weight of AP is 1250 (300–3000) [6].

### 4.3. Animals and Treatments

Male SHR and WKY (12 weeks old) were purchased from Japan SLC, Inc. (Shizuoka, Japan). The rats were housed at room temperature (24 ± 1 °C) and 55 ± 5% humidity with 12 h of light (artificial illumination: 08:00–20:00). After 1 week of prefeeding, the rats were divided into the following groups (*n* = 5 per group): (1) WKY-control group, (2) WKY-AP 3% group, (3) SHR-control group, (4) SHR-AP 1% group, and (5) SHR-AP 3% group. Each rat was then fed a normal diet or a normal diet containing 1%, or 3% AP ad libitum for 4 weeks. Systolic blood pressure (SBP), diastolic blood pressure (DBP), and heart rate were monitored by tail-cuff methods (BP-98A, Softron Co., Tokyo, Japan) every week. After 4 weeks, blood samples were collected, and the plasma was obtained by centrifugation. The medulla and cortex were isolated from the kidney and frozen in liquid nitrogen. The animals were trained for at least 2 days prior to the BP measurements in this study. All surgery was performed under diethyl ether anesthesia, and rats were euthanized by diethyl ether inhalation.

### 4.4. Measurement of ACE Activity

The tissue enzyme extracts of the organs were prepared by using Masuda and colleagues’ modified method [33,34]. Briefly, an organ was homogenized in 0.05 M HEPES (pH 7.9) containing 0.3 M NaCl with a Physcotron homogenizer (Microtec Co., Ltd., Chiba, Japan). The suspension was centrifuged (44,000× *g* for 90 min at 4 °C). The pellet was resuspended in buffer containing 0.5% Triton-X100. Each suspension was kept at 4 °C for 1 h and centrifuged (1000× *g* for 10 min at 4 °C). The resulting supernatant was used to measure ACE activity and protein content.

The plasma and tissue enzyme extracts were diluted with 0.1 M HEPES buffer (pH 8.3) containing 0.3 M NaCl and 0.01% Triton-X100. The ACE reaction was initiated by the addition of the ACE substrate hippuryl-histidyl-leucine, and the mixture was incubated at 37 °C for 30 min. After the reaction was stopped by the addition of 0.1 M NaOH, 1% *O*-phthalaldehyde in methanol was added. The mixture was maintained at room temperature for 10 min, and 0.1 M HCl was then added. After a 30-min incubation at 37 °C, the amount of liberated His-Leu was determined by measuring the fluorescence intensity of its adduct with *O*-phthalaldehyde (excitation at 390 nm and emission at 460 nm).

### 4.5. Measurement of SOD Activity

The levels of plasma SOD activity were measured using a SOD Assay Kit-WST, according to the manufacturer’s instructions.

### 4.6. Real-Time RT-PCR

RNA was extracted from the kidney with an RNeasy Mini Kit. A High Capacity cDNA Synthesis Kit was used to synthesize cDNA from 1 μg of RNA. Target gene expression was analyzed using real-time RT-PCR with the primers listed in Table 1. Real-time RT-PCR was conducted at a denaturation temperature of 95 °C for 15 s, an annealing temperature of 56 °C for 30 s, and an elongation temperature of 72 °C for 30 s. The amplification fluorescence intensity was monitored by using a MyiQ™ Single-Color Real-Time RT-PCR Detection System (Bio-Rad Laboratories). The mRNA gene expression levels were normalized to *18S rRNA* expression levels.

### 4.7. Statistical Analysis

Numerical data are expressed as the mean ± standard deviation. Significance was examined using Tukey’s test for multiple comparisons. Differences with a *p* < 0.05 were considered statistically significant.

## Figures and Tables

**Table 1:** Primer sequences for real-time PCR.

Target	Forward Primer (5′–3′)	Reverse Primer (5′–3′)
*SOD1*	TGTACCAGTGCAGGACCTC	ACACATTGGCCACACCGTC
*SOD2*	GACTGTGTTCCTGTGCACTG	GGATGACAGGAAGATGGTGAG
*p22*	GGCCTGATCCTCATCACAG	CAGATGAGCACTCCTGCAAC
*p47*	CAGGTGAAGAAGCCAGAGAC	CCCGATAGGTCTGAAGGATG
*18S rRNA*	GTCTGTGATGCCCTTAGATG	AGCTTATGACCCGCACTTAC

## Abbreviations

ACE	angiotensin-converting enzyme
AP	acacia polyphenol
DBP	diastolic blood pressure
NADPH	nicotinamide adenine dinucleotide phosphate
SBP	systolic blood pressure
SHR	spontaneously hypertensive rats
SOD	superoxide dismutase
ROS	reactive oxygen species
WKY	Wistar Kyoto rats
